# Incidence and predictors of tuberculosis occurrence among adults on antiretroviral therapy at Debre Markos referral hospital, Northwest Ethiopia: retrospective follow-up study

**DOI:** 10.1186/s12879-020-04959-y

**Published:** 2020-03-26

**Authors:** Agazhe Aemro, Abebaw Jember, Degefaye Zelalem Anlay

**Affiliations:** 1 Department of Medical Nursing, School of Nursing, College of Medicine and Health Sciences, University of Gondar, Gondar, Ethiopia; 2 Unit of Community Health Nursing, School of Nursing, College of Medicine and Health Sciences, University of Gondar, Gondar, Ethiopia

**Keywords:** Ethiopia, Incidence, HIV infection, TB/HIV infection

## Abstract

**Background:**

In resource limited settings, Tuberculosis (TB) is a major cause of morbidity and mortality among patients on antiretroviral treatment. Ethiopia is one of the 30 high TB burden countries. TB causes burden in healthcare system and challenge the effectiveness of HIV care. This study was to assess incidence and predictors of Tuberculosis among adults on antiretroviral therapy at Debre Markos Referral Hospital, Northwest Ethiopia, 2019.

**Methods:**

Institution based retrospective follow up study was conducted among adults on ART newly enrolled from 2014 to 2018 at Debre Markos Referral Hospital. Simple random sampling technique was used to select patients chart. Data was entered to EPI- INFO version 7.2.2.6 and analyzed using Stata 14.0. Tuberculosis incidence rate was computed and described using frequency tables. Both bivariable and multivariable Cox proportional hazard models was fitted to identify predictors of TB.

**Results:**

Out of the 536 patients chart reviewed, 494 patient records were included in the analysis. A total of 62 patients developed new TB cases during the follow up period of 1000.22 Person Years (PY); which gives an overall incidence rate of 6.19 cases per 100 PY (95% CI: 4.83–7.95). The highest rate was seen within the first year of follow up. After adjustment base line Hemoglobin < 10 g/dl (AHR = 5.25; 95% CI: 2.52–10.95), ambulatory/bedridden patients at enrolment (AHR = 2.31; 95% CI: 1.13–4.73), having fair or poor ART adherence (AHR = 3.22; 95% CI: 1.64–6.31) were associated with increased risk of tuberculosis whereas taking Isoniazid Preventive Therapy (IPT) (AHR = 0.33; 95% CI: 0.12–0.85) were protective factors of TB occurrence.

**Conclusion:**

TB incidence was high among adults on ART especially in the first year of enrollment to ART. Low hemoglobin level, ambulatory or bedridden functional status, non-adherence to ART and IPT usage status were found to be independent predictors. Hence, continuous follow up for ART adherence and provision of IPT has a great importance to reduce the risk of TB.

## Background

Tuberculosis (TB) is the most frequently diagnosed opportunistic infection among people living with human immunodeficiency virus (PLHIV). It remains one of the world’s leading causes of ill health and death among PLHIV [1–5]. Due to the immunological impairment associated to HIV infection, PLHIV are at higher risk of developing TB [6]. According to the 2017 global report, the risk of developing TB was 20 times higher in people living with HIV than in those without HIV [3, 7]. Globally, the life time risk of HIV positive individuals to develop TB is 50% and the annual risk is 10% [8].

From 2018 United Nations Program on HIV/AIDS (UNAIDS) global report, in 2017 there were 36.9 million people living with HIV. Of this 35.1 million were adults of age 15 year and above and about 940,000 people died from acquired immune deficiency syndrome (AIDS) related illnesses. TB accounts for around one in three AIDS related deaths [1] and from all TB/HIV related death about 84% is accounted in Africa [7].

Antiretroviral therapy has substantial potential to prevent HIV associated tuberculosis. So, in order to control HIV associated tuberculosis epidemic, earlier initiation of antiretroviral therapy may be a key component of global and national strategies [9]. Those patients who start ART earlier had a significantly higher rate of survival than those patients who started ART later [2].

The high burden of TB among patients accessing ART services in resource limited settings is a major cause of morbidity and mortality and is associated with nosocomial transmission risk which in turn results in multidrug resistant disease. Even though screening and diagnosis of TB is an essential component of patient care, it is challenging in HIV patients [10]. However, new diagnostic tools like gene Xpert provide a promising and more rapid diagnosis of TB in HIV patients [11].

Ethiopia is among the 30 high TB, HIV and multi drug resistant TB burden countries that accounted an overall annual TB incidence rate of 164 cases per 100, 000 populations [8, 12, 13]. Similarly, it is also one of the highly affected countries by the TB/HIV co-infection epidemic and associated with special diagnostic and therapeutic challenges resulting in extremely large burden on healthcare systems [14].

TB incidence ranged from 3.1–8.6 cases per 100 person year. A cohort study among adult HIV patients at Zewditu Memorial Hospital, followed from 2005 to 2009, showed that the incidence rate of TB was 3.1 cases per 100 person year [15]. Another 5 year retrospective follow up study conducted at Arbaminich General Hospital, from 2007 to 2013, showed that the incidence rate of TB was 5.36 cases per 100 person year [16]. The same study conducted in Afar public health facilities, Ethiopia, between 2010 and 2015 revealed that the overall incidence rate of TB was 8.6 cases per 100 person year [17]. Similar 5 year follow up study conducted at Gondar University Referral Hospital from 2007 to 2012 revealed the overall incidence rate of TB was 7.88 cases per 100 person year with high burden of TB incidence at the first year [18]. Therefore, the current study will show the evolution of the TB incidence over these studies.

Despite progress in care and prevention, the current speed of decline in the epidemiological burden of TB is not fast enough to reach targets set in the Sustainable Development Goals (SDGs). On the other hand, there is a global strategy known as “The End TB Strategy” aiming in reduction of TB incidence rate by 90% at the end of 2035. So, in order to ensure good progress of this strategy the current study has its own implication. Therefore, this study aimed to assess incidence and predictors of TB among adults on ART at Debre markos Referal Hospital.

## Methods

### Study design, setting and period

An institution based retrospective follow up study was conducted at Debre Markos Referral Hospital which is found in Debre Markos town. Debre Markos town is located in East Gojjam Zone of Amhara Nation Regional State [19]. It is situated 299 km from Addis Ababa, the capital city of Ethiopia, and 265 km from Bahir Dar, capital city of Amhara Nation Regional State [20]. Based on the 2007 national census conducted by the Central Statistical Agency (CSA) of Ethiopia, the population projection in 2012 Debre Markos has an estimated population of 262,497, of whom 129,921 were men and 132,576 women [19]. Debre Markos town has one referral hospital, four public health centers and seven private clinics. According to 2015 Debre Markos Referral Hospital human resource administration 3rd quarter report, the hospital was expected to serve more than five million people in the East and west Gojjam Zone.

Since 2005, the Hospital gives HIV care services both in outpatient and inpatient departments. Since the start of HIV care, the hospital has providing ART service for around 6, 350 patients who came from different areas and from this 5, 839 were adults. Of this, 1264 HIV infected adults were enrolled in to ART clinic between Jan 1, 2014 and Dec 31, 2018. There are 4 outpatient clinics such as 2 refill for adult, 1 refill for pediatric and 1 OIs treatment clinic. These unit provide ART service in the hospital with average visit of 36–40 patients to each unit per day [21]. Patient’s information’s were recorded both in electronic record systems and paper chart. Patients were monitored clinically, immunologically and virological but routine virological test was started since 2016. The hospital start test and start strategy since 2016.

### Inclusion criteria

All adults aged 15 years and above who were newly started ART and having at least one follow up at Debre Markose Hospital from Jan 1, 2014 to Dec 31, 2018 were included in this study. Whereas, those adults diagnosed as having TB and unrecorded for TB status at a baseline were excluded. Similarly, screening at the time of ART initiation was made and those diagnosed TB at the baseline were excluded from the study.

### Sample size and sampling procedure

Sample size was estimated by using single population proportion formula through EPI INFO statistical package version 7.2.2.6 with the assumption of 95% level of confidence, 2% marginal error, and by taking the incidence of TB 5.36% from previous study in Ethiopia (*Dalbo M, 2016)* [16]. With these assumptions the sample size became 487. Considering 10% expected incomplete record the final sample size was 536. Between January 1, 2014 and December 31, 2018 a total of 1264 adult patients were initiated ART and 1117 fulfill the inclusion criteria, of which 536 participants were selected. The patients’ chart number is taken from the electronic data base of the Hospital and 536 charts were selected through computer generated random number.

### Tuberculosis screening and diagnosis in HIV patient

All tuberculosis cases were diagnosed in Debre markos Hospital. Tuberculosis screening was done by using WHO screening tool (current cough of any duration, weight loss, fever, and night sweeting). Patients positive for a screening tool (at least positive for one of the symptoms) were investigated by bacteriological examination (gene Xpert and or culture). For those patients unable to produce sputum, difficult to get sample or negative result for bacteriological test other supportive investigations (histopathologic, radiologic and or ultrasonography examination) were done. According to the results of the investigation patients were classified as bacteriologically confirmed cases (If positive for bacteriological test) or clinical diagnosed cases (suggestive result by supportive investigation and decision made by expert clinicians).

### Operational definition

Incident TB case,which is an event of interest, in this study is defined asthe occurrence of TB cases after ART initiation which is bacteriologically confirmed (with at least one positive AFB microscopy, Xpert MTB/Rif assay positive, or culture positive) or based on Clinical decision of expert clinician by analyzing the supportive evidences (suggestive of TB) during follow up [22]. Patients with the first date of lost, drop out, transfer out, died or completed follow up period before developing tuberculosis were considered as censored. Level of adherence to ART drug in this study is classified as, Good (≥95% adherence or missing 1 out of 30 doses or missing 2 out of 60 doses), Fair (85–94% adherence or missing 2–4 out of 30 doses or missing 4–9 out of 60 doses), Poor (less than 85% or missing ≥5 doses of 30 doses or ≥ 10 dose out of 60 doses) [23]. Functional status of patients were classified according to the WHO criteria as working(W) capable of going out of home and do routine activities including the daily work, Ambulatory (A) capable of self-care and going to the toilet unsupported, Bed-ridden(B) cannot go even to the toilet unsupported. Patients are assigned to a particular stage when they demonstrate at least one clinical condition in that stage’s criteria. Patients remain at a higher stage after they recover from the clinical condition which placed them in that stage [22].

### Data collection tools and procedures

Data extraction tool was developed from HIV/AIDS care monitoring and evaluation tools. The tool contains socio-demographic, baseline clinical and treatment related variables. Since it was based on secondary data, forms used for laboratory request, ART intake forms and patient charts were reviewed. Training was given for data collectors regarding each description of the tool and the way they collect data from patient chart using the extraction tool. Data were collected by three nurses who had experience on HIV care. A medical record number of patients on the study period were taken from computer records in order to pick charts from patient chart room. Then data was collected from the patient’s medical charts by using prepared extraction tool. In order to avoid unintentional recollection of data, a commonly agreed code by data collectors was used. By this way all selected patient charts which fulfill the inclusion criteria were reviewed and data extraction was completed.

### Data quality control

To maintain data quality, data extraction tool was pre-tested for consistency and completeness of data items on 5% of patient charts. Data extraction process was monitored closely by the supervisor throughout the data collection period. The data clerks also assisted the data collectors by identifying the charts. After filling each extraction tool, completeness was checked and correction was made before the chart was returned back.

### Data processing and analysis

Data was checked for its consistency and completeness. It was coded and entered to EPI- INFO version 7.2.2.6 and exported to STATA version 14.0 for analysis. Statistical summary was applied to describe socio demographic, clinical and follow up variables of the study. TB Incidence rate for the study period was calculated and described by using frequency tables. The rate was calculated by dividing number of new TB cases occurring during the follow up period to total follow up time in year and expressed as per 100 person year. To estimate TB free survival probability, Kaplan Meier was used. Log rank test was also used to test equality of survivor. Model fitness was checked by using schoenfield residual test (*p*-value = 0.419) and goodness of fit was checked by using Cox-Snell residual test. After applying bivariable analysis, those variables with p-value < 0.2 was entered to multivariable Cox-proportional hazard model to identify predictors of TB incidence rate. 95% CI of hazard ratio was computed and variables having *p* value < 0.05 in the multivariate Cox proportional hazards model was considered as significantly and independently associated with incident tuberculosis.

### Ethical consideration

Ethical clearance was obtained from the Institutional Review Board (IRB) of the University of Gondar *(Ref. No.:-S/N/1600/06/2011).* Together with the ethical clearance informed consent waiver was obtained. Letter of cooperation was gained from school of nursing to collect data. To retrieve the data from the patients’ record permission was obtained from Debre Markos Hospital Medical director and ART focal person. To ensure confidentiality, personal identifiers like name were not registered in the extraction tool.

## Results

### Baseline socio demographic characteristics of study participants

Out of all patients enrolled to the ART clinic from January 1, 2014 to December 31, 2018, a total of 536 charts were selected and reviewed based on the inclusion criteria. From those reviewed charts, 494 were included in the analysis and the remaining 42 (7.84%) charts were excluded due to data incompleteness.

From 494 charts included in the analysis, around 303(61.3%) were females and about 200(40.5%) from the total subjects were in the age group of 25 to 34 years. The median age for this cohort was 33 years (IQR: 27–40 years). Almost all of the patients 472 (95.6%) were orthodox Christian and 171 (34.6%) of the total patients had no formal education. More than three forth 377 (76.3%) were urban dwellers and 228 (46.2%) of the participants were living with the family number of 3–4 members. A total of 457 (92.5%) patients had disclosed their HIV status. Of these 228 (46.15%) disclosed their status to their wife or husband.

### Incidence of tuberculosis

A total 494 patients included in the study followed up to 5 years, which gives a total 1000.22 person year observation. Study subjects were followed for a minimum of 0.93 month and a maximum of 59.43 months. The median follow up period was 21.77 months (IQR: 7.27–37.7). During the total follow up period, 62 new TB cases were observed which yielded a proportion of 12.55% (95% CI: 9.89, 15.79) and an overall incidence density rate of 6.19 cases per 100 person year (95% CI, 4.83, 7.95). Of the total incident TB cases, 49(79%) patients had Pulmonary TB while 13 (21%) had Extra pulmonary TB (EPTB) cases. Six (9.68%) of patients were diagnosed clinically.

Among the incident TB cases diagnosed within the 5 years follow up period, 29 were males and 33 were females. From all new TB cases during the follow up, 25 subjects (40.3%) were within the age group of 35–44 years. Twenty four (38.7%) of new TB cases had no any formal education and most (82.3%) of TB cases were from a family size of three or more individuals (Table 1).

**Table 1:** Socio-demographic characteristics and Tuberculosis incidence density rate stratified by socio-demographic characteristics of adults on ART at Debre-Markos Referral Hospital from January 1, 2014 to December 31, 2018 (*n* = 494)

Characteristics	Frequency (*n* = 494)	Percent (%)	Person year	TB (*n* = 62)	Censored (*n* = 432)	TB IDR per 100 PY (95% CI)
Age (years)						
15–24	61	12.35	120.43	6	55	4.99 (2.24, 11.09)
25–34	200	40.49	418.92	21	179	5.01 (3.27, 7.69)
35–44	160	32.39	314.16	25	135	7.96 (5.38, 11.78)
> 45	73	14.78	146.72	10	63	6.82 (3.67, 12.67)
Sex of patient						
Male	191	38.66	362.88	29	162	7.99 (5.55, 11.50)
Female	303	61.34	637.35	33	270	5.18 (3.68, 7.28)
Marital status						
Single	62	12.55	95.32	11	51	11.54 (6.39, 20.84)
Married	267	54.05	572.27	33	234	5.77 (4.09, 8.11)
Divorced/separated	129	26.11	271.63	12	117	4.42 (2.51, 7.78)
Widowed	36	7.29	61.01	6	30	9.83 (4.42, 21.89)
Educational status						
No education	171	34.60	357.45	24	147	6.71 (4.50, 10.02)
Primary	113	22.90	215.81	19	94	8.80 (5.62, 13.80)
Secondary & above	210	42.51	426.96	19	191	4.45 (2.84, 6.98)
Occupation						
Has job	159	32.19	250.92	16	143	6.38 (3.91, 10.41)
Jobless	109	22.06	201.25	19	90	9.44 (6.02, 14.80)
Not recorded	226	45.75	548.05	27	199	4.93 (3.38, 7.18)
Residence						
Urban	377	76.32	777.71	45	332	5.79 (4.32, 7.75)
Rural	117	23.68	222.51	17	100	7.64 (4.75, 12.29)
Family size						
≤ 2	197	39.88	372.33	11	186	2.95 (1.64, 5.33)
3–4	228	46.15	475.49	43	185	9.04 (6.71, 12.19)
≥ 5	69	13.97	152.39	8	61	5.25 (2.63, 10.49)
HIV Disclosure Status						
Disclosed	457	92.51	927.87	55	402	5.93 (4.55, 7.72)
Not Disclosed	37	7.49	72.35	7	30	9.68 (4.61, 20.29)
Total	494	100	1000.22	62	432	6.19 (4.83, 7.95)

From the total study participants, 432 (87.45%) patients a counted for censored. Of which, 7 (1.62%) lost from follow up, 21(4.86%) drop, 7 (1.62%) dead, 77 (17.82%) transferred to other health facility, and 320(74.08%) were on follow up to the end of the study without developing tuberculosis.

### Baseline and follow up clinical and immunological characteristics of study participants

Out of the total 494 study participants with complete information for analysis, majority (97.17%) of them had not TB treatment history. A total of 219 (44.3%) patients were in baseline WHO clinical stage I. Only 38 (7.7%) of all patients had baseline CD4 count of less than 50 and majority were found in a group with CD4 count ≥350 cells/μl. The median base line CD4 count was 314.5 cells/μl (IQR: 147–443). Three hundred and fifty-nine patients (72.7%) were in the baseline hemoglobin level of ≥10 g/dl and the median hemoglobin level was 13.2 g/dl (IQR: 11.3–14.5).

Almost two third (67.4%) of participants were having a baseline body mass index of ≥18.5 kg/m^2^ with median BMI of 20.58 kg/m^2^ (IQR: 18.29–23.01). A total of 392 (79.4%) patients were enrolled to chronic HIV care clinic with working functional status. Only 79 (16%) patients had past opportunistic infections and from these only 14 patients (2.83%) had history of TB. Majority of patients, 467 (94.5%) were on TDF-3TC–EFV ART regimen at the baseline and only 16 (3.2%) of the participants were changed their regimen during follow up. Of those who have changed their initial regimen during follow up more than half (56.3%) were due to treatment failure. From all patients, 127 (25.7%) had fair or poor ART adherence level during follow up and 299 patients (60.5%) did not take IPT. More than one third (36.4%) of patients were followed for about 1–3 years.

From sixty two incident TB cases, TB were observed higher on patients with base line WHO clinical stage III (14.06 cases per 100 PY; 95% CI: 10.18, 19.40) and stage IV (26.37 cases per 100 PY; 95% CI: 14.97, 46.43). It was also observed that, TB incidence was noticeable among patients with baseline CD4 count of < 50 cells/μl (18.77 cases per 100 PY; 95% CI: 11.31, 31.13) and being from 50 to 100 cells/μl (18.93 cases per 100 PY; 95% CI: 11.93, 30.04). Patients with baseline hemoglobin < 10 g/dl was higher incidence, 20.17 cases per 100 PY (95% CI: 15.37–26.47). Of new TB cases, three fourth of cases had a BMI < 18.5 kg/m^2^ having an incidence 16.32 cases per 100 PY (95% CI: 12.26–21.72).

Forty seven patients having baseline ambulatory or bedridden functional status developed TB during follow up with an incidence of 28.00 cases per 100 PY (95% CI: 21.04, 37.27). From those new TB patients during the follow up, 47 cases were fair or poor ART adherence contributed for an incidence rate of 19.89 cases per 100 PY (95% CI: 14.95, 26.48). An incidence of 9.61 cases per 100 PY (95% CI: 7.41, 12.45) was observed on those patients who did not take IPT during follow up period. The highest incidence of TB was seen within the first year of follow up (58.13 cases per 100 PY; 95% CI: 43.11–78.37) (Table 2).

**Table 2:** Tuberculosis incidence density stratified by clinical and immunological characteristics of PLHIV on chronic HIV care at Debre-Markos Referral Hospital from January 1, 2014 to December 31, 2018 (N = 494)

Characteristics	Frequency (*n* = 494)	Percent (%)	Person year	TB (*n* = 62)	Censored (*n* = 432)	TB IR per 100 PY (95% CI)
Past TB treatment						
Yes	14	2.83	36.14	6	8	16.60 (7.46, 36.95)
No	480	97.17	964.08	56	424	5.81 (4.47, 7.55)
Baseline WHO clinical staging						
Stage I	219	44.33	396.81	7	212	1.77 (0.84, 3.70)
Stage II	124	25.10	294.67	6	118	2.04 (0.91, 4.53)
Stage III	122	24.70	263.23	37	85	14.06 (10.18, 19.40)
Stage IV	29	5.87	45.51	12	17	26.37 (14.97, 46.43)
Baseline CD4 count (cells/μl)						
< 50	38	7.69	79.93	15	23	18.77 (11.31, 31.13)
50 – < 100	51	10.32	95.09	18	33	18.93 (11.93, 30.04)
100–200	75	15.18	170.31	14	61	8.22 (4.87, 13.88)
201–350	123	24.90	268.43	10	113	3.73 (2.00, 6.92)
≥ 350	207	41.90	386.46	5	202	1.29 (0.54, 3.11)
Baseline hemoglobin level (g/dl)						
< 10	135	27.33	257.82	52	83	20.17 (15.37, 26.47)
≥ 10	359	72.67	742.41	10	349	1.35 (0.72, 2.50)
Baseline body mass index (kg/m^2^)						
< 18.5	161	32.59	288.03	47	114	16.32 (12.26, 21.72)
≥ 18.5	333	67.41	712.19	15	318	2.11 (1.27, 3.49)
Baseline functional status						
Working	392	79.35	832.36	15	377	1.80 (1.09, 2.99)
Ambulate/bedridden	102	20.65	167.86	47	55	28.00 (21.04, 37.27)
Past opportunistic infection (OIs)						
Yes	79	15.99	215.17	18	61	8.37 (5.27,13.28)
No	415	84.01	785.05	44	371	5.60 (4.17, 7.53)
ART adherence						
Good	367	74.29	763.97	15	352	1.96 (1.18, 3.25)
Fair/poor	127	25.71	236.25	47	80	19.89 (14.95, 26.48)
Took IPT during follow up						
Yes	195	39.47	406.90	5	190	1.23 (0.51, 2.95)
No	299	60.53	593.32	57	242	9.61 (7.41, 12.45)
Total year of follow up						
≤ 1 year	169	34.21	73.98	43	126	58.13 (43.11, 78.37)
> 1 - < 3 years	180	36.44	353.47	11	169	3.11 (1.72, 5.62)
3–5 years	145	29.35	572.78	8	137	1.39 (0.69, 2.79)

### Kaplan-Meier plot for TB-free survival probability among adults on ART

**Fig. 1:**
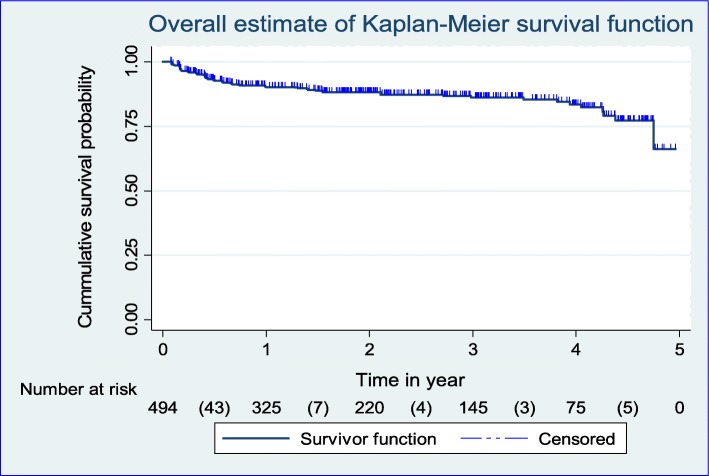
Kaplan-Meier curve of TB-free survival probability among cohorts of PLHIV on chronic HIV care at Debre-Markos Referral Hospital from January 1, 2014 to December 31, 2018

TB-free survival probability for the total cohort at the end of 6 month was 0.93 (95%CI; 0.90, 0.95); at the end of 12 month was 0.90 (95%CI; 0.87, 0.93); at the end of 2 years was 0.88 (95%CI; 0.85, 0.91); at the end of 3 year was 0.86 (95%CI; 0.82, 0.89); at the end of 4 year was 0.83 (95%CI; 0.78, 0.88); and that of surviving TB free at the end of follow up was 0.66 (95%CI; 0.41, 0.83) (Fig. 1).

**Fig. 2:**
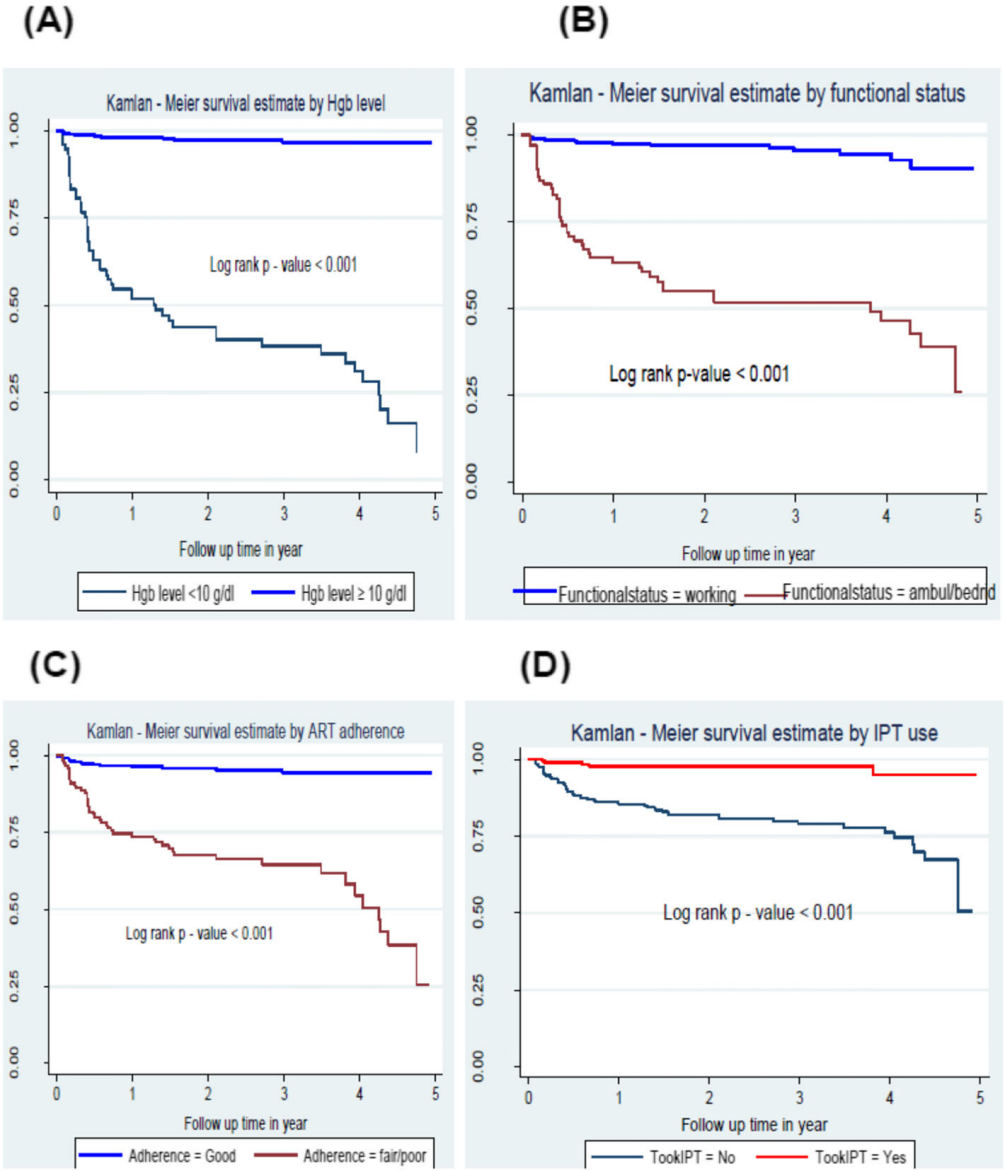
Kaplan-Meier plots of TB-free survival probability: (**a**) stratified by baseline Hgb level (**b**) by functional status; (**c**) by ART adherence; (**d**) by IPT use among PLHIV on chronic HIV care at Debre-Markos Referral Hospital: Jan 1, 2014 to December 31, 2018

Log rank test of equality of survival for baseline Hemoglobin level, Functional status, ART adherence level and IPT usage were significantly associated with TB incidence rate of people living with HIV. Patients whose baseline hemoglobin level less than 10 g/dl had a significantly lower TB-free survival probability compared to those with hemoglobin ≥10 g/dl. Those patients who had baseline functional status of working, used IPT during follow up and had good ART adherence had significantly higher TB free survival probability than those patients with ambulatory or bedridden functional status, didn’t use IPT and had fair or poor ART adherence level respectively (Fig. 2).

### Predictors of tuberculosis occurrence

In the bivariable Cox regression analysis, past TB treatment history, baseline CD4 count, WHO clinical stage at baseline, baseline haemoglobin level, baseline BMI, functional status at baseline, HIV disclosure status, ART adherence during follow up, and IPT use were found to be predictors of the incidence of TB among PLHIV at a *P*-value of less than 0.2. These variables were also checked for Cox proportional hazard assumption and they didn’t violate the assumption. So, they were selected for multivariable Cox-regression analysis. From these, baseline haemoglobin level, functional status at enrolment, IPT use and ART adherence level were found to be statistically significant predictors of TB incidence rate among adults on ART at a P - value of less than 0.05 (Table 3).

**Table 3:** Cox regression analysis of predictors of tuberculosis among cohorts of PLHIV on chronic HIV car at Debre-Markos Referral Hospital from January 1, 2014 to December 31, 2018 (*n* = 494)

Variable	Survival status		CHR (95% CI)	Adjusted	
	Event	Censored		AHR (95% CI)	*P*-value
Past TB treatment					
Yes	6	8	2.84 (1.22, 6.64)	1.01 (0.38, 2.69)	0.981
No	56	424	1.00	1.00	
HIV disclosure					
Disclosed	7	30	0.59 (0.27, 1.30)	0.74 (0.31, 1.76)	0.495
Not disclosed	55	402	1.00	1.00	
WHO staging					
I/II	13	330	1.00	1.00	
III	37	85	7.55 (4.01, 14.22)	1.27 (0.59, 2.71)	0.534
IV	12	17	13.37 (6.09, 29.36)	2.18 (0.86, 5.52)	0.100
CD4 count (cells/μl)					
≤ 200	47	117	11.28 (4.48, 26.36)	2.31 (0.85, 6.30)	0.101
201–350	10	113	3.04 (1.04, 8.89)	1.34 (0.43, 4.17)	0.608
≥ 350	5	202	1.00	1.00	
Hemoglobin level					
< 10 g/dl	52	83	14.41 (7.32, 28.38)	**5.25 (2.52,10.95)**	< 0.001*
≥ 10 g/dl	10	349	1.00	1.00	
BMI (kg/m^2^)					
< 18.5	47	114	7.51 (4.19, 13.44)	1.97 (0.99, 3.88)	0.052
≥ 18.5	15	318	1.00	1.00	
Functional status					
Working	15	377	1.00	1.00	
Ambulatory/bedridden	47	55	14.10 (7.87, 25.28)	**2.31 (1.13, 4.73)**	0.021*
ART adherence					
Good	15	352	1.00	1.00	
Fair /poor	47	80	9.76 (5.45, 17.47)	**3.22 (1.64, 6.31)**	0.001*
Took IPT					
Yes	5	190	0.13 (0.05, 0.31)	**0.33 (0.12, 0.85)**	0.023*
No	57	242	1.00	1.00	

* Independently significant predictors of TB at *p*-value < 0.05

## Discussion

Diagnosis of HIV-associated TB infection in the era of ART has always been a difficult task and remains the major public health problems in many parts of the world. In this study, the overall incidence rate of TB among adults on ART is 6.19 cases per 100 PY (95% CI: 4.83–7.95). This finding is in line with TB incidence rate reported from Arbaminich general hospital which was 5.36 cases per 100 PY and university of Gondar referral hospital which was 7.88 cases per 100 PY. This similarity with the current study could be due to comparable sample size, equal length of follow up duration (i.e. 5 years); even the study settings were almost similar regarded to facilities and service provision [16, 18].

Lower TB incidence rate was observed in the current study than that of Afar public health facilities which was 8.6 cases per 100 PY [17]. This might be that, in the current study, TB/HIV treatment guidelines were changing; hence, many patients have initiated ART earlier which justifies treatment as prevention [9]. Improvement in TB/HIV care service integration, better awareness of the population regarding TB/HIV co-infection prevention might also contribute for the decrement [24, 25]. Moreover, an increase in global and national concern towards TB/HIV co-infection than the previous years and difference in the socio economic status between Afar and Debre markos might contribute for the observed difference in TB incidence.

In contrary, TB incidence rate in the current study was higher than that of Zewditu memorial hospital (3.1 cases per 100 PY), Addis Ababa, Ethiopia. Zewdu hospital is the first public hospital in Ethiopia that begins to give HIV care services for patients and it is known by the quality of care delivered for PLHIV. So, this might contribute for the lower incidence of tuberculosis [15].

In the current study, the highest incidence rate of TB was observed in the first year of enrolment which was 58.13 cases per 100 PY (95% CI: 43.11, 78.37). This indicates that the highest probability of developing TB case after starting ART was observed sooner. This study was also congruent with other studies conducted at Afar public health facilities [17] and University of Gondar referral hospital [18]. This peak TB incidence shortly after enrollment might be due to ART induced unmasking of latent TB phase or new infection. Missing ART dose due to ART drug side effect nearly after enrolment might also contribute the high TB incidence at the first year of follow up [26].

This study showed that patients whose baseline hemoglobin level less than 10 g/dl were 5.25 times (AHR = 5.25; 95% CI: 2.52–10.95) at higher risk of developing TB at any time as compared to patients with hemoglobin level of greater than or equal to 10 g/dl. This is in line with previous studies conducted at Arbaminchi general hospital [16] and Addis Ababa [27]. This might be due to the direct effect of HIV and its viral proteins on bone marrow stem cell results in bone marrow suppression which might cause anemia and the probability of predicting the occurrence of TB becomes high [28]. This shows the need for earlier initiation of treatment to control and reduce virus number and prevent opportunistic infections by boosting immunity.

Patients with baseline ambulatory or bedridden functional status were 2.31 times at risk of developing TB as compared to patients with working functional status at enrolment to ART (AHR = 2.31; 95% CI: 1.13–4.73). This finding is agreed with other studies conducted in Afar public health facilities (AHR = 2.42), University of Gondar Hospital (AHR = 1.64) and Amhara regional state (AOR = 1.73) [17, 18, 29]. This is due to the fact that ambulatory or bedridden functional status is an indicator of low immune status and the presence of associated opportunistic infections, including TB [30].

Individuals who took IPT during follow up were 67% less likely to develop TB as compared with those who did not take IPT (AHR = 0.33; 95% CI: 0.12–0.85). This might be due to the synergist role of IPT and ART drug in reducing the incidence of TB among PLHIV. Introduction of IPT to those patients on ART reduces the mycobacterium load which in turn reduces the progression of latent TB to active one [31–33]. This finding is consistent with those studies in Ethiopia [17, 27, 34, 35]. This entails the need for provision of IPT for all eligible patients on HAART to prevent TB.

Level of adherence to ART is also the predictors of incident TB to occur. Patients with fair or poor ART drug adherence were 3.22 times at risk of developing TB than patients with good ART drug adherence (AHR = 3.22; 95% CI: 1.64–6.31). This study was in line with a previous study conducted in Harari, Eastern Ethiopia [36]. This might be due to the fact that, adherence problem in ARV drug might hinder the effect of ART drug on viral load suppression which in turn results in waning of CD4 cell count. Therefore, the patient became more susceptible to different opportunistic infections including TB.

Due to its retrospective nature of the study few patient record with incomplete data were excluded from the analysis which might underestimate or biased the result if the lost was related to TB. In addition, some important variables like viral load, monthly income and status of substance use were not included in this study.

## Conclusion

Incidence of tuberculosis was high among adults on ART especially in the first year of enrollment to ART. Low hemoglobin level, baseline ambulatory/bedridden functional status, ART adherence level and IPT usage status were found to be independent predictors of tuberculosis occurrence among adults on ART. Hence, tuberculosis prevention strategies especially in the first year of enrollment among PLHIV need to be strengthened by ensuring optimum provision of Isoniazid (INH). Continuous monitoring of ART adherence and resolve a problems early is helpful to prevent symptomatic tuberculosis. Further researches with prospective cohort study by incorporating important variables which could be difficult to extract retrospectively like the effect of viral load and socioeconomic variables can give full insight on the distribution of factors for the occurrence of tuberculosis in the region.

## Data Availability

The datasets used and/or analysed during the current study available from the corresponding author on reasonable request.

## Abbreviations

3TC: Lamivudine
AFB: Acid Fast Bacilli
AHR: Adjusted Hazard Ratio
AIDS: Acquired Immunodeficiency Syndrome
ART: Antiretroviral Therapy
AZT: Zidovudine
BMI: Body Mass Index
CD4: Cluster of Differentiation Four
CHR: Crude Hazard Ratio
CI: Confidence Interval
COR: Crude Odds Ratio
CPT: Cotrimoxazole Prophylactic Therapy
CSA: Central Statistical Agency
EFV: Efavirinz
Hgb: Hemoglobin
HIV: Human Immunodeficiency Virus
IDR: Incidence Density Rate
INH: Isoniazid
IPT: Isoniazid Preventive Therapy
MDR: Multi Drug Resistant
NVP: Nevirapine
OIs: Opportunistic Infections
PLHIV: People Living With Human Immunodeficiency Virus
PY: Person-year
SDGs: Sustainable Development Goals
TB: Tuberculosis
TDF: Tenofovir Disoproxil Fumarate
UNAIDS: The Joint United Nations Program on HIV/AIDS
WHO: World Health Organization
